# A Systems-Genetics Approach and Data Mining Tool to Assist in the Discovery of Genes Underlying Complex Traits in *Oryza sativa*

**DOI:** 10.1371/journal.pone.0068551

**Published:** 2013-07-16

**Authors:** Stephen P. Ficklin, Frank Alex Feltus

**Affiliations:** 1 Plant and Environmental Sciences, Clemson University, Clemson, South Carolina, United States of America; 2 Department of Genetics & Biochemistry, Clemson University, Clemson, South Carolina, United States of America; New Mexico State University, United States of America

## Abstract

Many traits of biological and agronomic significance in plants are controlled in a complex manner where multiple genes and environmental signals affect the expression of the phenotype. In *Oryza sativa* (rice), thousands of quantitative genetic signals have been mapped to the rice genome. In parallel, thousands of gene expression profiles have been generated across many experimental conditions. Through the discovery of networks with real gene co-expression relationships, it is possible to identify co-localized genetic and gene expression signals that implicate complex genotype-phenotype relationships. In this work, we used a knowledge-independent, systems genetics approach, to discover a high-quality set of co-expression networks, termed Gene Interaction Layers (GILs). Twenty-two GILs were constructed from 1,306 Affymetrix microarray rice expression profiles that were pre-clustered to allow for improved capture of gene co-expression relationships. Functional genomic and genetic data, including over 8,000 QTLs and 766 phenotype-tagged SNPs (*p*-value < = 0.001) from genome-wide association studies, both covering over 230 different rice traits were integrated with the GILs. An online systems genetics data-mining resource, the GeneNet Engine, was constructed to enable dynamic discovery of gene sets (i.e. network modules) that overlap with genetic traits. GeneNet Engine does not provide the exact set of genes underlying a given complex trait, but through the evidence of gene-marker correspondence, co-expression, and functional enrichment, site visitors can identify genes with potential shared causality for a trait which could then be used for experimental validation. A set of 2 million SNPs was incorporated into the database and serve as a potential set of testable biomarkers for genes in modules that overlap with genetic traits. Herein, we describe two modules found using GeneNet Engine, one with significant overlap with the trait amylose content and another with significant overlap with blast disease resistance.

## Introduction

The past century has seen major advances in our understanding of genotype-phenotype relationships underlying Mendelian and complex traits controlled primarily by large-effect genes. However, methods for discovery of the genetic factors controlling complex traits are not fully mature, limiting our ability to use genetic-based methods for understanding some diseases and for breeding of certain traits in plants and animals. In plants such as *Oryza sativa* (rice), quantitative trait loci (QTL) mapping analysis has been a key method for identifying genomic positions associated with traits of interest. While QTL mapping analysis has been successful in associating some traits with large-effect genes [1], [2], it has failed to identify the genetic factors for traits comprised primarily of small-effect genes. In a 2009 review on the status of QTL analysis for rice, Yamamoto *et. al.* suggest the need for integration of genomics-based methods to improve the sensitivity for discovery of small-effect genes [3]. Association mapping studies such as recent Genome-wide Association Studies (GWAS) studies for rice [4], [5] offer greater potential for finding QTLs with large and small-effect genes but in both cases, identification of the underlying genes, as well as the functional network in with they participate may not be known. Gene co-expression networks, integrated with genetic data (e.g. from QTL mapping, GWAS), and functional genomic information, offer the potential to identify gene sets underlying complex traits. This combination of network biology, genetics and genomics data is a recent area of study known as systems genetics [6], [7].

Gene co-expression networks, or relevance networks [8], [9], are increasingly common tools that describe complex gene expression relationships. Co-expression networks consist of a set of nodes interconnected by edges. In gene co-expression networks genes are nodes and edges (or lines) connect two nodes when their expression levels are significantly correlated across a set of expression measurement samples (e.g. Pearson’s correlation coefficient (PCC)). Co-expression networks have specific topological properties similar to most naturally occurring networks: they are often scale-free, hierarchical and small world [10]. Typically, construction of gene co-expression networks uses microarray-derived expression profiles as input, although RNA-seq datasets have recently been used [11], [12]. A wealth of publicly available expression datasets are currently available in repositories such as the NCBI Gene Expression Omnibus (GEO; [13]), Short Read Archive (SRA; http://www.ncbi.nlm.nih.gov/Traces/sra/), ArrayExpress from the European Bioinformatics Institute [14], and other sources. The samples submitted to these repositories (e.g. microarrays and RNA-seq datasets) include a record of the experimental conditions (i.e. genotype, environment, tissue, developmental stage). After network construction, highly-connected genes are circumscribed into gene modules. Modules are sets of nodes that tend to be more highly connected amongst themselves than with other nodes in the network. Nodes within a module tend to be involved in similar biological processes, therefore, modules that contain genes with no known function can be ascribed putative function through “guilt-by-association” inferences [8], [15]. Many co-expression networks for plants are currently available [16], [17], [18], [19], [20], [21], [22], [23], [24], [25], [26], [27], [28], [29]. Also, the utility of co-expression networks has spurred development of numerous online web resources available for exploration of gene interaction relationships in plants [22], [23], [25], [26], [30], [31], [32], [33], [34].

A deepening view of gene output captured in public expression profiles can be mined to build as holistic a view as possible of gene interaction for an organism. Typically, when co-expression networks are constructed input samples (such as microarrays or RNA-seq datasets) are either segregated using a knowledge-dependent method [29], [35] or combined into a single input set [23], [25], [34]. However, there are limitations to both approaches for maximal discovery of an organism’s interactome. Segregating samples using a knowledge-dependent approach relies on human knowledge, and sometimes imprecise and inconsistent vocabularies to identify conditions. Even for highly controlled experiments, unknown variables in each sample set increase noise within the dataset, thus limiting capture of co-expression relationships. Combining all samples into a single compendium exacerbates the problem, especially as the sample set contains measurements from a highly diverse set of conditions [36]. While a completely holistic, “pan” co-expression network is not possible (as we cannot measure every gene in every experimental condition), improved, knowledge-independent methods are needed to detect co-expression relationships for all conditions using smarter dataset sorting approaches.

Therefore, the objective of this work was to build a high resolution series of rice gene co-expression networks using an optimized RMTGeneNet network construction pipeline [37] to bring a high-level, holistic view of the interaction space of rice*–*one of the most important staple food crops in the world. Knowledge-independent methods for network construction and module discovery were employed to overcome knowledge-bias in the detection of rice gene interaction. Prior to co-expression network construction, we used K-means clustering of input microarray samples. This approach attempts to maximize capture of gene interactions that otherwise would be hidden in noise if all samples were used as a single input set. Our approach generated multiple co-expression networks from the full set of Affymetrix GeneChip® Rice Genome arrays available in NCBI GEO at that time–one for each K-means cluster. We refer to each network as a Gene Interaction Layer (GIL). Using this improved capture of gene co-expression in the GIL collection, we aimed to integrate genetic data from QTL mapping experiments and Genome Wide Association Studies (GWAS) to highlight network modules with potential quantitative phenotype association. To help explore the rice GIL collection and associated genetic signals, we created a new online data mining resource called GeneNet Engine for exploration of network modules with potential association to genetic traits. Genes within significant network modules serve as potential candidates underlying complex genetic traits and potentially contain small effect genes.

## Results

### Network Construction

Prior to network construction, 1,306 microarrays were downloaded from NCBI GEO [13] and pre-processed including normalization, outlier detection and removal of control and ambiguous probesets. Ambiguous probesets are those that map to more than one locus on the rice genome. In total, 123 control probesets and 4,772 ambiguous probesets were removed, as well as 19 outlier microarrays. Microarrays were then clustered into 25 groups using K-means clustering (Hartigan and Wong implementation from the *kmeans* function of the R statistical package [38]). K-means is a cluster analysis method that groups input microarrays into *k* sets in such a way that the sum of squares within the group is minimized. Thus for the microarrays used for this project, the 25 groups contained microarrays whose expression level at each probeset were most similar to others in the group. A co-expression network for each K-means cluster was then constructed using the RMTGeneNet package [37]. RMTGeneNet first generates pair-wise Pearson Correlation Coefficients (PCC) for all genes and then uses Random Matrix Theory (RMT) [39] to identify an optimal threshold for culling PCC values. Of the 25 clusters, the RMT method generated 22 co-expression networks, or Gene Interaction Layers (GILs). Three clusters failed to generate networks. One cluster had fewer microarrays than a required cutoff of 25, and the RMT method failed to identify a threshold for two others. The number of input microarrays per GIL ranged from 19 to 231 with an average size of 53.8 and a median of 39 (Table 1). The probesets of the input microarrays of each GIL were mapped to 46,498 of the 57,133 genes (81%) on the Michigan State University’s (MSU) v6.0 Rice genome [25]. The collection of GILs contains 282,484 edges among 16,664 nodes (genes) and together captures 35% of the measurable genes of the array and 29% of the total genes of the MSU v6.0 genome. For all GILs, the PCC threshold was quite high, ranging from 0.91 to 0.99 indicating that all relationships (edges) are highly co-expressed.

**Table 1:** Network details from *k*-means clustered microarray samples into 25 groups.

Network	Input Arrays	Outlier Arrays	Total Edges	Total Nodes	RMT Threshold	<*k*>^a^	Modules
G0001	22	0	*Failed to construct: RMT could not find threshold*				
G0002	81	0	19,338	2,890	0.91	13.38	569
G0003	73	2	36,346	3,155	0.92	23.04	676
G0004	14		*Failed to construct: too few arrays*				
G0005	26	0	14,641	1,991	0.97	14.71	290
G0006	32	0	38,331	1,914	0.97	40.05	355
G0007	90	0	12,059	2,806	0.91	8.60	370
G0008	65	3	18,383	3,276	0.91	11.22	476
G0009	25	0	3,579	1,366	0.99	5.24	173
G0010	16	1	*Failed to construct: RMT could not find threshold*				
G0011	74	0	10,411	1,624	0.96	12.82	397
G0012	40	0	29,971	3,622	0.93	16.55	522
G0013	37	0	2,366	896	0.98	5.28	150
G0014	118	3	6,738	1,963	0.90	6.87	330
G0015	21	0	9,374	1,034	0.98	18.13	256
G0016	21	1	2,358	1,670	0.97	2.82	129
G0017	24	0	8,688	1,607	0.96	10.81	194
G0018	36	0	8,434	1,660	0.95	10.16	216
G0019	19	1	4,689	3,022	0.97	3.10	234
G0020	73	3	6,268	2,308	0.91	5.43	260
G0021	231	2	227	204	0.98	2.23	24
G0022	58	0	7,516	2,007	0.92	7.49	279
G0023	54	3	34,398	1,596	0.94	43.11	302
G0024	39	0	5,880	2,512	0.95	4.68	325
G0025	57	0	2,489	1,167	0.98	4.27	135
Total	1,346	19	282,484	n/a^b^			6,662

Microarrays are from the NCBI GEO platform GPL2025.

a The average degree of a GIL.

b The total number of nodes is 16,664 across all GILs and nodes may be present in multiple GILs.

### Gene Module Detection and Co-Similarity

**Figure 1:**
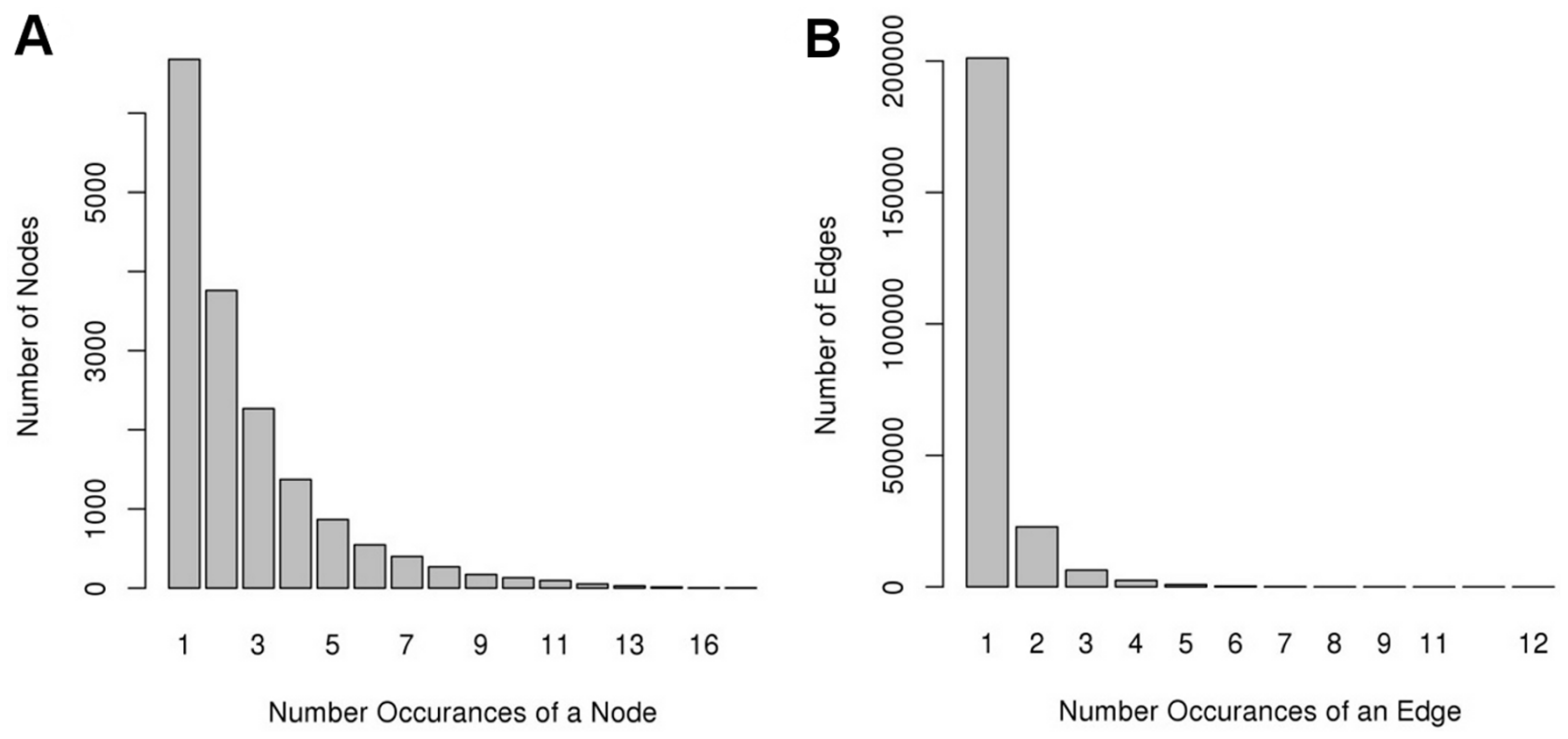
Redundant Edges and Nodes. The number of times that a A) rice gene (node) or B) co-expressed gene pair (edge) appears in different GILs.

The link community method [40] was used to find modules, or sets of nodes (genes) that are more highly connected with each other than with the rest of the network. The link community algorithm was employed using the linkcomm R package [41]. The method allows for nodes to be present in more than one module thus supporting the theory that genes can be multi-functional. In total, 6692 link community modules (LCM) were discovered. Modules were named using a three-part schema separated by an underscore (e.g. OsK25v1.0_G0011_LCM020), where the first part ‘OsK25v1.0’ represents the *O. sativa* GIL collection version 1.0 (derived from presorting with K-means 25), a second part prefixed with the letter ‘G’ indicates the GIL to which the module belongs and the third part prefixed by ‘LCM’ indicates the unique module within the GIL. The average number of modules per GIL was 302.8 and the median 284.5 (Figure 1). The collection of GILs represents interactions between 35% of the measurable genes and some of those genes are present in more than one GIL. As shown in Figure 1A, the majority of nodes are present in only a single GIL (6,608 nodes, 40%), and the number of times a node appears in multiple GILs decreases. Edges tend to be more unique per GIL as 201,121 (71%) are only found in a single GIL and the number of times an edge appears in more than one GIL is significantly less (Figure 1B).

**Figure 2:**
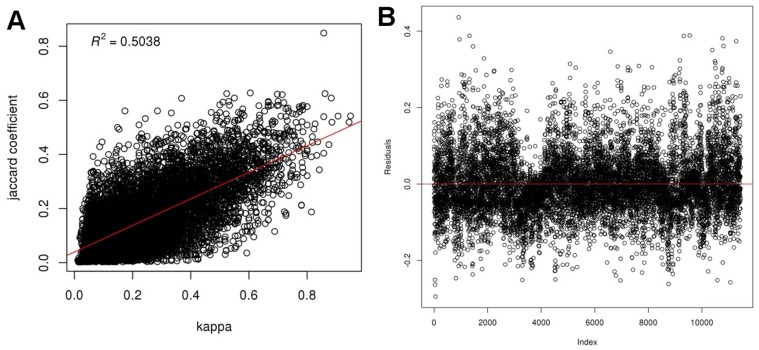
Jaccard vs Kappa Scatterplot. Jaccard (similarity of node composition) and Kappa (similarity of functional annotation) statistics were performed, pair-wise, for all modules across all GILs. A) The scatterplot of Jaccard coefficient vs Kappa κ for all modules with 30 or more nodes. B) Residual plot of Jaccard coefficient vs Kappa κ.

To obtain a measure of similarity between modules across all GILs, a correlation between Kappa scores (measuring functional similarity between two modules) and Jaccard indices (measuring similarity of node composition) was performed. First, functional enrichment analysis of the modules was performed using terms from the Gene Ontology (GO; [42]), InterPro [43] and KEGG [44]. Only terms enriched within a module with a Fisher’s *p*-value of 0.01 or less were considered enriched. Next, full pair-wise comparisons between modules with 30 or more nodes from all GILs were performed using both Kappa statistics and a Jaccard similarity test. Only enriched functional terms were used with the Kappa test. Kappa scores range from −1 to 1 with values less than 0 indicating no significant similarity of function and a score of 1 indicating identical similarity of function. A Jaccard index ranges from 0 (indicating no nodes in common) to 1 (all nodes in common). Figure 2 shows a scatterplot of Jaccard similarity coefficients versus Kappa scores with *R* ^2^ = 0.5 (*p*-value <2.2e-16) indicating a good degree of correlation between the node composition of modules and the enriched function of modules.

**Figure 3:**
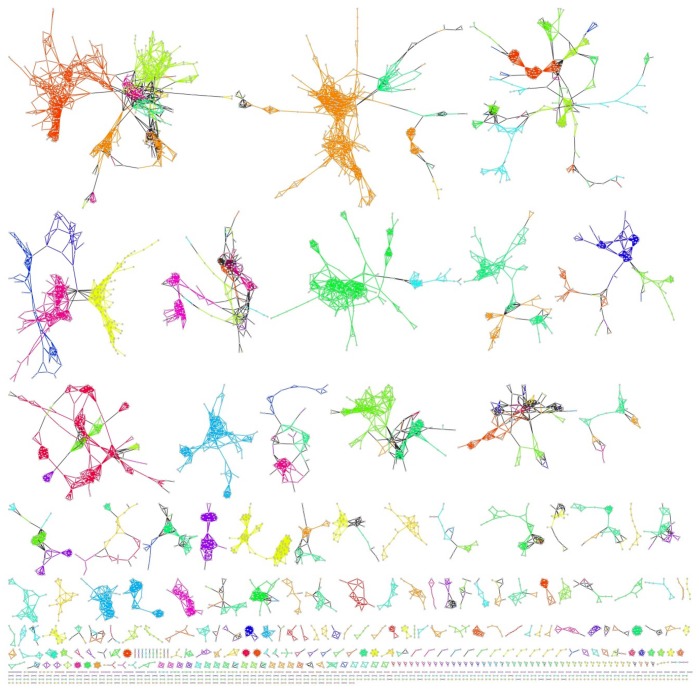
Gene Module “meta-network”. The nodes in the meta-network are LCM modules from all GILs that have a pair-wise Kappa score > = 0.5 and Jaccard coefficient > = 0.3. Edges are colored if both nodes in the edge belong to the same GIL. Each GIL is assigned a unique color. Edges where each node belongs to a different GIL are black. Nodes are grey.

A meta-network of LCM modules was then created using the similarity scores as described previously. In theory, a Kappa score greater than 0 can be considered meaningful however in practice higher values are often used for greater stringency. We used a Kappa score threshold of 0.5, which corresponds to a Jaccard score of approximately 0.3 in the scatterplot of Figure 2. Edges were added to the meta-network between pairs of modules with a Kappa score of 0.5 or greater. Figure 3 shows a diagram of the LCM module meta-network. In this network, the nodes are LCM modules and edges indicate a high degree of similarity (Kappa >0.5 and Jaccard >0.3). The edges are color-coded according to the GIL to which the modules belong. If two modules from different GILs shared an edge, then the edge is black. The meta-network contains 13,578 edges across 4,965 LCM modules (75% of all LCM modules). The number of edges in the meta-network that connect LCM modules of the same GIL is 12,253 (90%) with 1,325 (10%) connecting two different GILs.

### Interactive Systems-Genetics Exploration Tool

To integrate genetic data with GILs, and to construct an online resource for exploration of genotype-phenotype relationships, the physical positions of significant genetic data from QTLs and GWAS studies were obtained. Over 8,000 QTL intervals, along with their corresponding genomic coordinates were downloaded from Gramene’s QTL database [45]. Also a 300 kb LD window surrounding significant SNPs (*p*-value <0.0001) from a recent GWAS study by Zhao *et al* were used [4]. Genes overlapping both QTL and GWAS SNP intervals were putatively assigned the trait. These associations as well as all GILs were input into an online database called GeneNet Engine which is available online at http://sysbio.genome.clemson.edu. The data is housed in a Chado database schema [46] with custom tables and visualized using Tripal [47]. Next all available rice SNPs from NCBI’s dbSNP [48] database were uniquely mapped to the rice genome and loaded into the database so that an end-user can identify proximal biomarkers for genotype-phenotype hypothesis testing. Users can query the database using a locus name, module name, functional term, or trait of interest to examine the possibility that one or more modules may play a role in a particular function. Supplemental Figure S1 provides a screen shot of the search engine.

**Figure 4:**
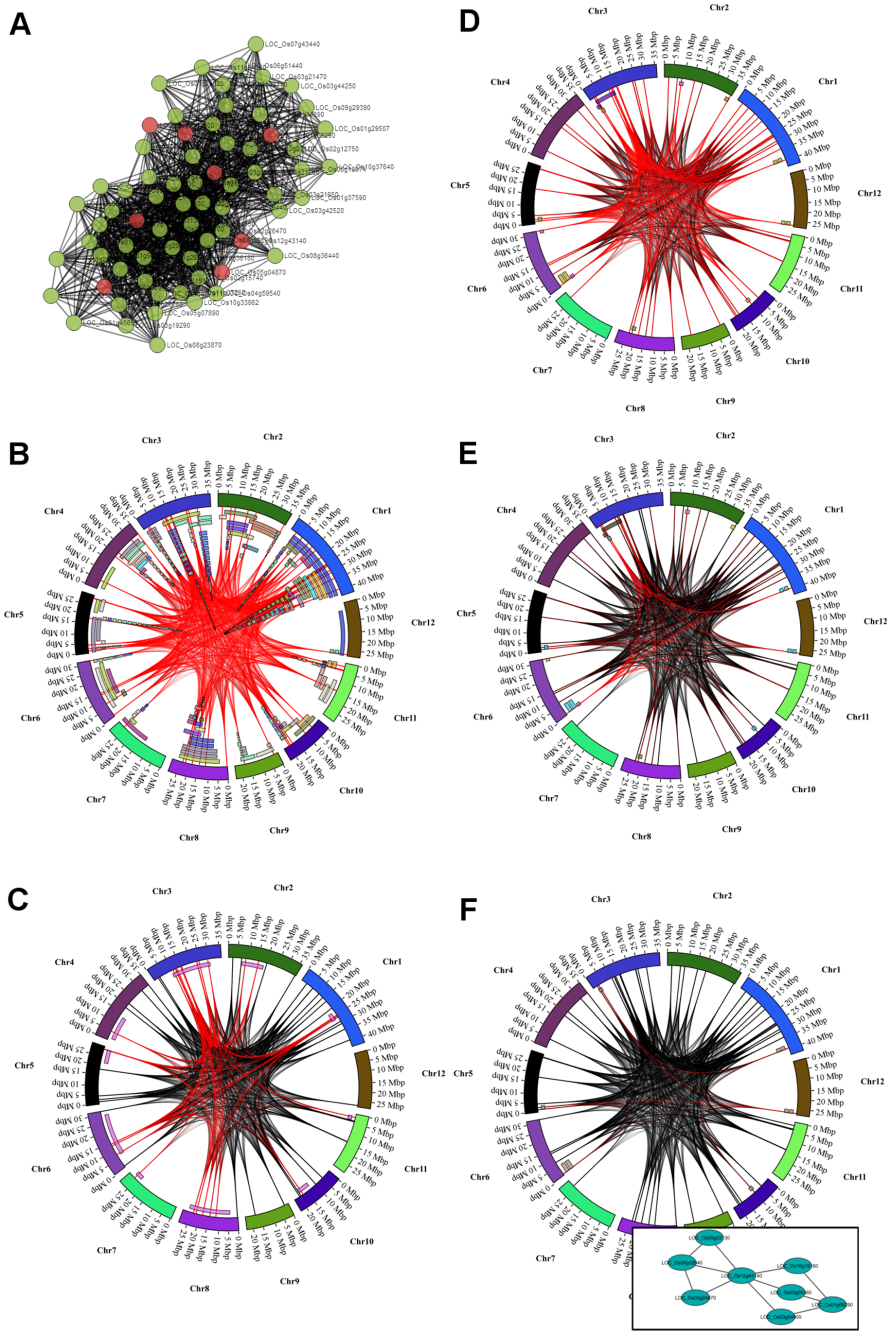
Circular Genome Plots of Network Module OsK25v1.0_G0002_LCM0431. The chromosomes of rice are shown as the outer circle. Gray arcs are edges of the module. Endpoints of each edge are fixed on the physical location in the genome where the node (gene) is found. Red arcs are edges that overlap a genetic feature. The colored tiles along the chromosomes represent genetic features (e.g. QTLs or regions around significant SNPs in GWAS). A) Network view of the module. Red nodes overlap with genetic traits for amylose content, green nodes do not. B) Circular plot of the module with all genetic features for plant height. C) Plot with QTLs from a single genetic map (Cornell 9024/LH422 RI QTL 1996) and edges highlighted red where an edge overlaps at least two QTL. D) Plot of the all genetic features for amylose content where edges overlap with at least 1 genetic feature. E) Plot of all genetic features for amylose content with overlap of at least 3 genetic features. F) Plot of module edges with QTLs from a single genetic map (CNHAU Zhen97/H94 QTL 2005) with overlap of at least 3 genetic features. The inset graph shows the connectivity of the overlapping nodes.

The GeneNet Engine also provides a module explorer. The module explorer (Supplemental Figure S5) consists of a set of tabs that provides network visualization (‘Module View’ tab), a genome network visualization (‘Genome View’ tab), lists of module nodes, edges, functionally enriched terms, a form for specifying traits to select (‘Filter by Trait’ tab), a list of all overlapping traits and genetic features, and a form for generating a list of potential SNP biomarkers that flank highlighted nodes within a specified window size. In the network module view, an interactive module is provided using Cytoscape Web [49]. Users are presented a network module with which they can move nodes, and zoom in and out. Clicking a node will provide functional annotations about the node (locus details box in Supplemental Figure S2). In the ‘Filter by Trait’ tab, users can dynamically alter the module view or genome view by selecting one or more specific traits, a genetic feature type (e.g. QTL or GWAS SNP) and by limiting the number of overlapping traits an edge must pass through to be highlighted (Supplemental Figure S3). Additionally, circular plots are available in the ‘Genome View’ tab allowing visitors to visualize the network within the context of the chromosomal coordinates as well as visualization of QTL or GWAS SNP regions that overlap with nodes in the module. Examples of circular plots for the module OsK25v1.0_G0002_LCM0431 can be seen in Figure 4. For reference, the module view is present in Figure 4A. Figures 4B–F highlight changes in the genome view as filtering parameters are changed. Figure 4B shows overlapping edges with QTLs for plant height. Edges with at least one node within a QTL region are colored red. In cases where there are large QTLs or where QTLs cover large swaths of the genome, almost all of the edges are red. Figure 4C shows the same plot but only with a single QTL set for plant height. These QTL are all from the same genetic map and fewer overlaps are present. Figure 4D shows the same module overlapping genetic features for the trait amylose content. While not as dense as QTLs for plant height they do overlap a large portion of the module. Therefore, a limit that an edge must pass through at least 3 different genetic features was imposed for the image in Figure 4E. Figure 4F contains the plot for amylose content overlapping QTLs from a single genetic map. Users can obtain *p*-values for the filters they employ by looking on the ‘Genetic Features’ tab of the Module Explorer.

## Discussion

The primary objectives of this project were three-fold. The first objective was to use all publicly available microarray-based RNA expression profiling data in NCBI GEO to generate co-expression networks for *O. sativa* that could capture as many gene interactions as possible. Second, was to integrate, on a massive scale, network nodes with results from genetic analyses such as QTL mapping experiments and GWAS studies with the expectation that network modules could serve as a genome reduction strategy for finding genes that may be associated with a given trait. The final objective was to construct a systems genetics data mining platform for discovery of relationships between network modules and genetic traits and the reagents that could be readily used for hypothesis testing.

One major challenge mentioned in the Introduction was that of overcoming an increase in noise due to increases in conditions under which gene expression is measured. Performing a gene pair-wise correlation across all input samples only allows for genes that are similarly expressed across all conditions to be found. Gene correlations expressed in only a few microarrays will not be found due to dilution. A larger and more diverse input dataset would result in a smaller network [36]. Additionally, thresholding methods such as *ad hoc* methods [8], [50], [51], [52] have been used to allow for flexible thresholding but, they provide little statistical guidance and can incorporate non-significant relationships. To capture all relationships in the dataset, we avoided methods that require bait genes, such as linear regression [16]. Rank-based methods [9], [15] did offer an attractive feature in that they allow for dynamic thresholding. Dynamic thresholding does not apply a constant threshold across the entire set of PCC values, but rather examines the neighborhood around each gene to determine a local threshold. Partial Correlation and Information Theory (PCIT) [36] and supervised machine learning [53], [54] also generate high-quality networks with dynamic thresholding, but were not currently adaptable to our network pipeline. By pre-clustering of microarrays based on gene expression pattern alone, we are able to use Random Matrix Theory (RMT) to provide thresholding for a highly significant set of relationships for each GIL. A unique RMT threshold is determined for each GIL, thus our approach behaves similarly to a dynamic thresholding method but without dependence on global PCC values such as the case with rank-based methods. Because RMT is knowledge-independent and is not biased towards prior and possibly incomplete knowledge, we were able to capture a very high quality set of relationships derived solely on the underlying expression values. While we used K-means clustering for pre-sorting, any number of data clustering methods could be used.

**Figure 5:**
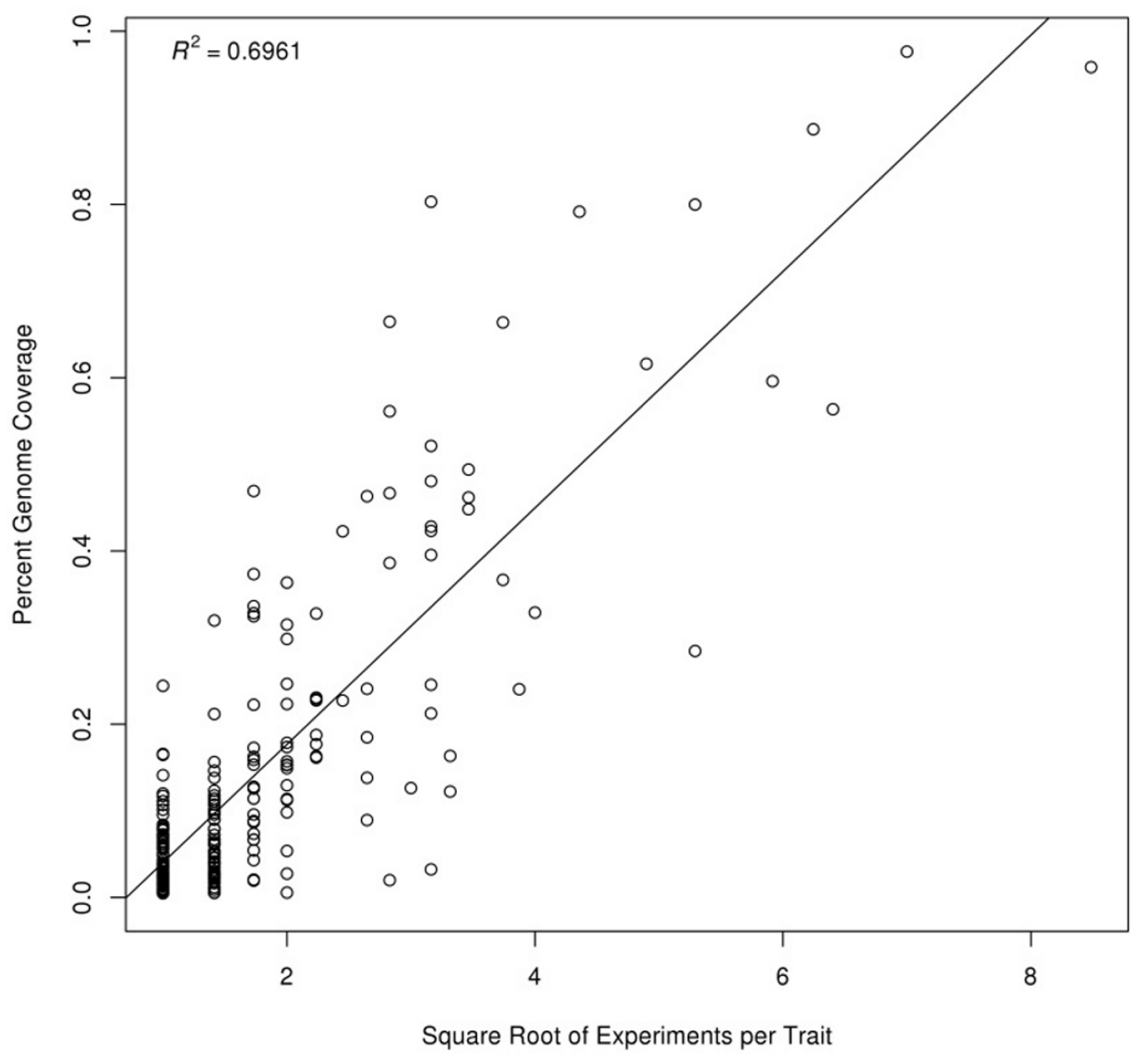
Number of QTLs per Trait vs Genome Coverage. The scatterplot shows the relationship between the total percent covered of the physical genome versus the square root of the number of experiments per trait for QTL data from Gramene. Inset shows plot of residuals.

The availability of rich genetic data for rice was a key motivation for this study. We used approximately 8,000 genome mapped QTLs from Gramene. The Gramene curators painstakingly mapped markers for all QTLs to the MSU v6.0 genome assembly, thus providing genomic coordinates for the QTLs. The precise genes causal for many of the traits underlying these QTLs are unknown. Therefore, we simply assigned the QTL trait to all genes underlying the QTL intervals. Given the imprecision of QTL mapping, and our assigning a trait to all genes underlying a SNP or QTL region, we introduce many false positive gene-phenotype associations. The visualizations and lists provided on the GeneNet Engine (Figures 5 and 7) will highlight all genes and edges from a network module that overlap with a QTL or GWAS SNP, but most likely will include false positives by random chance alone. The probability that a network module could contain a gene underlying a region for a genetic feature can be quite high, especially in the case of large QTLs, many QTLs for the same trait or where the module is large. Additionally, other factors such as tandem array genes (TAGs) can bias correspondence *p*-values due to overlap redundancy. TAGs typically are involved in similar function or pathways and hence would be co-expressed and typically present in the same module. TAGs therefore would bias *p*-values calculations that expect a normal distribution. Despite these challenges we simply provide a Fisher’s test as a probability metric for false positives. However, we caution that this is only meant as a guide for filtering modules of interest, and further work is needed to identify an appropriate method for *p*-value calculation.

Because we mixed microarrays with probesets mostly derived from *O. sativa spp japonica* we obtained network relationships likely to be enriched for the japonica subspecies as a whole and not specifically for a single genotype. However, the microarray platform has been used for multiple subspecies and varieties of rice. Therefore, it may be possible that a network module may represent pathways specific to an individual or subspecies, and other modules could be specific to other subspecies. Moreover, a module could be a conglomeration of interactions across a set of individuals or subspecies. As evidence for this, a linear relationship exists between the square root of the number of QTLs (across all studies) and the amount of genome space they cover (Figure 5). This seems to confirm the notion that hundreds (or potentially thousands) of genes may contribute to a trait, and as more genotypes are analyzed, the more genes that are captured by QTLs. The GWAS study by Zhao *et. al.* also suggests that different groups of genes control the same trait in different subpopulations [4]. Therefore, it would seem that the collection of all QTLs for a given trait becomes an approximation of a pan-QTL set for the species. Similarly, the GIL collection is an approximation of a pan co-expression network.

**Figure 6:**
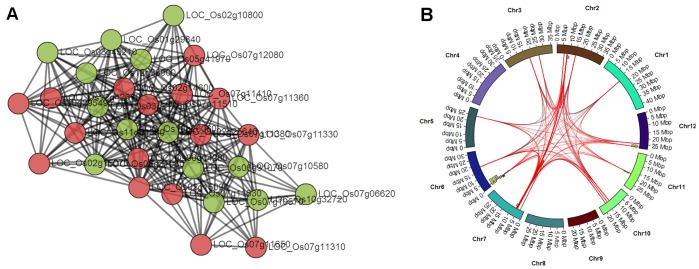
A Significant Module for Amylose Content. Module OsK25v1.0_G0023_LCM0301 significantly overlaps with 15 different genetic features (2 SNPs, 13 QTLs, *p*-value = 1.9e-4) and is significantly enriched for Bifunctional trypsin/alpha-amylase inhibitor helical domain and starch synthase. A) Red circles indicate nodes that overlap with genetic features and green nodes do not. B) The distribution of module edges along the genomic chromosomes. GWAS SNPs are barely visible as tick marks whereas QTLs are visible as small colored blocks along the chromosomes. Edges are red if one node lies within the region of a genetic feature.

To demonstrate the use of the GeneNet Engine, we use as an example the trait amylose content. It is well understood that the Waxy gene (Wx) plays a major role in amylose content [55]. This gene resides on chromosome 6 of *Oryza sativa* and is at locus LOC_Os06g04200 on the MSU v6.0 genome. A recent study of 171 rice accessions shows that two SNPs in the Waxy gene account for 86.7% of the variation in amylose content [56], indicating it is a large effect gene. Recently, Zhao *et. al.* included amylose content as a trait in their GWAS study and significantly identified 68 SNPs associated with amylose content with a mixed model *p*-value <1e-4 [4]. In an effort to find small effect loci that may affect variation in amylose content, a search was performed using the GeneNet Engine. Using the search page a filter was entered that provided the Waxy gene locus, LOC_Os06g04200, as well as overlap with the amylose content trait. In this case, the genetic feature was limited to a ‘GWAS SNP’. The result yielded 6 modules from the Rice GIL collection and one from a previous global rice network [25] which has also been added to the GeneNet Explorer. Most of the network modules were small (between 5–15 nodes). In the GIL collection, the largest module was OsK25v1.0_G0023_LCM0301, with 30 nodes, and it had the largest average connectivity (<*k*> = 17.47) indicating that the nodes were more highly interconnected than the other 5 modules. The GeneNet Engine provides a Fisher’s *p*-value as a simple means for filtering modules that may have a high probability of false positives. As mentioned previously, this *p*-value is simply a guide and does not necessarily imply a high probability of causality for the trait. The top enriched functional terms for all 7 modules included seed storage protein (IPR006044), alpha-amylase inhibitor (IPR013771), and transcription factor CBF/NF-Y (IPR003958). All 6 GIL collection modules were present in GIL G0023 except for one (enriched for Transcription factor CBF/NY-Y) which was present in GIL G0003. Starch synthase (K00703) was also enriched in all 7 modules. All 6 of the Rice GIL modules overlapped with only 1 or 2 GWAS SNPs, with *p*-values quite high (from 0.2 to 0.03), indicating a high probability of false positives. However, after including overlapping genes underlying QTLs using the ‘Filter by Trait’ tab in the Module Explorer, the *p*-values were all lower and the most highly connected GIL module, OsK25v1.0_G0023_LCM0301, overlapped with 13 QTLs and 2 GWAS SNPs (15 genetic features) with a *p*-value of 1.9e-4 (Figure 6). The module from the global network was much larger, overlapped 4 GWAS SNPs and 34 QTLs but had a high probability of false positives (*p*-value = 0.03). While p-values were not significant for some of the smaller modules, it would seem that any of these modules could be potential candidates to explore small-effect variation in amylose content. Potentially, combining several of these modules may provide, as a group, a set of possible small-effect candidate genes. The OsK25v1.0_G0023_LCM0301 module seemed most suited for exploration as it is relatively small (only 30 genes) had a significant *p*-value (1.9e-4) and all nodes were highly connected indicating a high degree of cooperation. The effects of these genes may be examined through additional lab experiments, such as where plants with mutations can be grown and phenotyped. As a direct means for verification through experimentation, GeneNet Explorer can provide a list of SNPs that could potentially serve as biomarkers. For module OsK25v1.0_G0023_LCM0301, over 4200 SNPs were obtained, all within 50 kb of genes that overlapped genetic features for amylose content.

**Figure 7:**
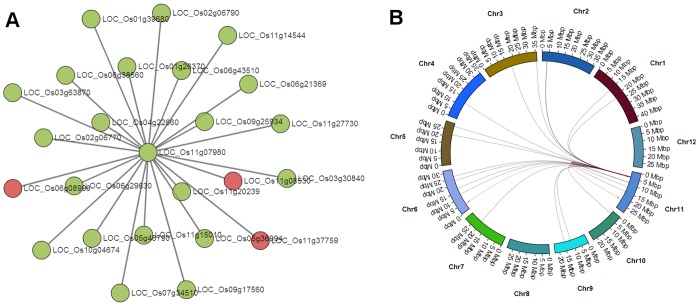
A Significant Module for Blast Disease Resistance. Module OsK25-v1.0_G0008_LCM0015 significantly overlaps with 3 different GWAS SNPs (*p*-value = 5.9e-4) and is functionally enriched for Ankyrin, Syntaxin and disease resistance protein. A) Red circles indicate nodes that overlap with genetic features and green nodes do not. B) The distribution of module edges along the genomic chromosomes. GWAS SNPs are barely visible as tick marks and edges are red if one node overlaps the region surround a GWAS SNP.

As a second example we use the trait for blast disease resistance. The Pi-ta gene is known to be associated with blast resistance [57]. The locus for this gene on the MSU v6.0 genome is LOC_Os12g18360, but unlike the example for amylose content, it does not appear in any network modules. Additionally, 200 QTLs are present for blast disease resistance which covers a large portion of the genome. Therefore, the chance that any module would overlap with the set of QTLs for blast disease resistance is high. However, only 16 GWAS SNPs were associated (mixed model *p*-value <0.0001). Therefore, a search was entered into GeneNet Engine to find modules overlapping with the blast disease resistance trait but only that overlapped with GWAS SNPs. Additionally, a limit of 10 nodes was included to limit the appearance of smaller modules. A total of 242 matching modules were returned. Results were sorted by an increasing node size and examined to find modules overlapping more SNPs than other modules of similar size. The module OsK25-v1.0_G0008_LCM0015 had a module size of 25 nodes and overlapped with 3 SNPs while others of similar size overlapped with 1 or 2. Figure 7 shows the network view and genome plot for this module which has a false positive Fisher’s *p*-value of 5.9e-4. The module is enriched primarily for an Ankyrin repeat (IPR002110), but also for Syntaxin (IPR006011, IPR006012, SNARE proteins) and for disease resistance protein (IPR000767). There are several Ankyrin repeat containing proteins that are involved in many biological processes but they are also known to participate in disease resistance, such as in the case of the *OsBIANK1* gene which is expressed during infection of *Magnaporthe grisea,* the blast disease fungus [58], [59]. Additionally, Syntaxin SNARE has been shown to participate in resistance to pathogens through membrane-vesicle fusion in the delivery of anti-pathogen compounds [57]. The evidence provided by the overlap of 3 module nodes with 3 of the 16 SNPs (*p*-value = 5.9e-4) associated with blast disease resistance in addition to the functional annotations make the OsK25-v1.0_G0008_LCM0015 a good candidate for further study of potential genes that participate in resistance to blast fungus infection. Any of the genes in this module could potentially serve as small effectors of the trait.

The methods for discovery of significant modules for both examples above were somewhat different. In the first example a known gene was used to guide discovery of interesting modules, whereas for the second a significant module was found by browsing through a few hundred results. As seen in Figure 4, a module can overlap with many genetic features from multiple traits (e.g. plant height and amylose content). This should be expected naturally as genes are known to be multi-functional, but most likely many of these overlaps are false positives. Therefore, the GeneNet Engine will calculate *p*-values for false positives dynamically as users change filtering parameters in the Module Explorer, thus allowing users to explore different filters. Also, as mentioned previously, the more experiments across genotypes the more likely the QTLs will cover more of the genome, creating more false positives raising *p*-values for all modules that overlap with the trait. In these cases, users may want to focus on modules that overlap with individual genetic maps. Users can filter by genetic map in the ‘Filter by Trait’ tab of the GeneNet Explorer (Supplemental Figure S3). Therefore, it may be necessary to apply various searching approaches to find modules of interest for a specific trait, but as demonstrated in the two examples, interesting modules for further testing can be found.

The rice K-means 25 GIL collection and the GeneNet Engine are the first release of a large-scale, integrated systems-genetic resource for plants to help with prediction of genes underlying complex traits. However, several improvements can be made. The choice of a *K* value of 25 was selected by using the common “rule of thumb” function of *k* = √(*n*/2). However, we were only able to capture 35% of the measurable genes on the Affymetrix GeneChip array. This fell short of our goal to capture near 100% of the measurable genes; however this level of coverage is possible. In another study where the approach of pre-clustering was applied to *Arabidopsis thaliana*, approximately 98% of genes were capture in the GIL set (unpublished data). For that study, a *K* value was selected by iterating through different *K* sizes to maximize gene capture. It would be beneficial to find a more appropriate value of *K* for constructing a rice GIL collection that captured relationships from more genes in the array, with the potential of capturing all of them. Alternatively, other more dynamic pre-clustering methods may be used other than *K*-means to improve interaction capture.

Additionally, it may be beneficial to augment module detection to take into account overlap with genetic features. For this project we used the link community method for module discovery [40]. This method and many others rely on parameter settings that can be more or less inclusive. Therefore, network modules are a function of not only the underlying connectivity but the parameters used during execution of the algorithm. Generating modules that optimally capture a specific biological process is challenging and one set of parameters may capture well some processes but not others. In the first example for amylose content, all 6 modules overlapped with genetic traits for amylose content, had the Waxy gene and all had similar functional enrichment. All modules were relatively small with the exception of the largest module, OsK25v1.0_G0023_LCM0301, and all modules, except one, came from the GIL G0023. This concurs with the fact that GILs tend have modules of similar function. As seen in the scatterplot of Figure 2 and the meta-network of Figure 3, network modules tend to be most similar to other modules within the same GIL. It would seem, therefore, that the module detection algorithm could potentially take advantage of genetic and functional relatedness to stitch together potentially more significant modules. But, in summary, a more flexible and dynamic module creation method may improve the creation and identification of gene sets underlying complex traits.

### Conclusion

Here we present the Rice GIL collection of networks that are a first attempt at using pre-clustering of *O. sativa* RNA expression profiles to capture all co-expression relationships measured by the full compendium of publicly available microarrays at NCBI GEO. Our goal has been to guide network construction and module discovery solely through the evidence of gene expression. The knowledge-independent approach reduces bias towards our limited knowledge of the underlying biological processes. We integrate experimentally validated genetic data from over 8,000 rice QTLs from Gramene and significant SNPs from a recent rice GWAS study to create a platform for discovery of network modules that may be associated with trait causality. The platform is made available in the form of an interactive website named GeneNet Engine found at http://sysbio.genome.clemson.edu. The value in this approach is two-fold. First, it brings to light potentially small-effect genes (those that are connected in the module) and serves as a filtering technique to locate genes that underlie genetic features for complex traits such as QTLs. We anticipate that significant or interesting modules from GeneNet Engine can be used for further lab-based experimentation which can translate to quicker discovery of genes underling complex traits and perhaps future application in rice breeding.

## Materials and Methods

### Construction of the Rice GIL Networks

Before construction of the Rice GIL networks, all available microarrays from the Affymetrix GeneChip® Rice Genome array were obtained from NCBI GEO [13]. At the time, 1306 were retrieved. All microarrays were then pre-processed with RMA normalization [60] using RMAExpress [61] and outliers were detected using the arrayQualityMetrics package [62] for BioConductor [63]. Microarrays that failed at least two of the three outlier test were removed. The output consisted of an *m*×*n* expression matrix where *m* is the number of micorarrays and *n* is the number of probesets on the array. Next, control probes were removed from the matrix as well as ambiguous probes that mapped to more than one gene.

After pre-processing the microarrays in the expression matrix were then grouped. The *kmeans* function of R (using the Harding and Wong implementation [38]) was used to segregate microarrays into sets where the sum of squares of each probeset is minimized. A value of *k* = 25 was determined using the common “rule of thumb” function of *k* = √(*n*/2), and hence 25 clusters of samples were generated. Twenty-two separate networks were then constructed by first passing each group through the same pre-processing, quality control pipeline described previously: samples within a group were normalized, outliers were removed and control and ambiguous probesets were removed. The construction method required that a network have at least 25 microarray samples. The list of microarray samples, the *K*-means cluster (and GIL) that each belongs to and characteristics of each sample are provided in Supplemental Table S1.

Next, the co-expression network for each *k*-means group was constructed using the RMTGeneNet software package [37]. RMTGeneNet is a software package written in the C programming language that quickly generate correlation matrices and network adjacency matrices. RMTGeneNet first performs pair-wise correlation analysis for every probeset on the array, generating an *m*×*m* similarity matrix of correlation values ranging from −1 to 1. Next, it employs Random Matrix Theory (RMT) [39] to find an optimal threshold. According to RMT, the more random a matrix, the more the nearest-neighbor spacing distribution (NNSD) of eigenvalues appears Gaussian. The less random, the more Poisson-like it appears. RMT determines a threshold for the similarity matrix by measuring when the NNSD ceases to appear Poisson (*p*-value = 0.001). An adjacency matrix is constructed by setting all values less than the threshold to zero. In total, 22 adjacency matrices were produced: one for each K-means cluster. Finally, probesets were mapped to genes in the MSU Rice v6.0 [64] assembly of the *Oryza sativa* genome, and 22 gene co-expression networks, or Gene Interaction Layers (GILS), were constructed. GILs were generated in parallel using Clemson University’s Palmetto computation cluster.

### Module Discovery

After construction of the 22 GILs, modules were determined using the link-community method [40]. This approach allows a gene to be present in multiple modules. This approach is more reasonable for multi-functional genes and does not restrict genes to a single module such as other methods (e.g. MCL [65]). We used the *linkcomm* function for R [41] to generate LCM modules for all 22 GILs.

### Functional Enrichment

All modules from all GILs underwent functional enrichment analysis to look for significantly over-represented terms in relation to the genomic background. Terms from the Gene Ontology [42], and InterPro [43] databases mapped to genes were obtained directly from the MSU website and KEGG [44] terms were mapped to genes using the KEGG Automatic Annotation Server [66]. Functional enrichment was performed using a DAVID-like [67], [68] Perl script developed in-house. Terms enriched with a Fisher’s test *p*-value <0.01 where kept.

### Genome Mapping of Genetic Data

Genetic data from the Gramene QTL database [45], [69], and from a recent GWAS study [4] were used in this study for associating traits with network modules. Gramene curators used marker information to map over 8,000 QTL regions from various studies to positions on the *Oryza sativa* MSU v6.0 genome sequence. We then putatively associated all genes underlying the QTL regions the QTL trait. QTLs that only mapped to a single marker and were therefore smaller than 5 bp were enlarged to 2 Mb (the median size of QTLs from Gramene). For the GWAS study, only significant SNPs (*p*-value <0.0001) from the mixed model analysis were used. Genes within a 300 kb window around the SNP were putatively associated with the SNP trait. The range of 300 kb flanking was used because this was the estimated average linkage disequilibrium for *Oryza sativa japonica* reported in the GWAS study (the largest of the three subspecies). The trait names used for both the QTLs and SNPs are from Gramene’s Trait Ontology (TO) [70]. The TO terms used for both QTLs and SNPs were provided by Gramene. Additionally, traits from the *Tos*17 retrotransposon study [71], [72] were also included in this study but were associated to network modules using the same process as for functional enrichment described previously. The process was the same as described for the global network for *Oryza sativa* [25]. The gene assignments to *Tos*17 phenotypes as well as enrichment are present in the GeneNet Engine but are not discussed in this manuscript.

### Data Storage and Visualization

All genomic, genetic and network data was stored within a Chado database [46]. Custom tables were created for storing network data (nodes, edges, and modules). Materialized views were constructed to enable faster searching. Visualization of genomic, genetic and network data was implemented using Tripal [47], an open-source publicly available construction toolkit for online genomic and genetic databases. A custom Tripal extension module was written specifically for this project and used for display of network data, as this functionality was not already part of Tripal. Cytoscape Web [49] was used for the network module visualization and the d3 JavaScript library (http://d3js.org) was used for drawing the circular genome plots. Network modules from all 22 GILs are searchable on the GeneNet Engine v0.9 site at http://sysbio.genome.clemson.edu. The Tripal Network extension module is freely available, but is under active development and is therefore available upon request.

### Use of SNPs

SNPs from NCBI’s dbSNP database [48] for *Oryza sativa* were obtained through bulk download from dbSNP’s FTP site. SNPs were then mapped to the MSU v6.0 build of the *Oryza sativa* genome using blat [73]. Only SNPs that mapped once to the genome with a minimum percent identity of 0.98 across the full length of the SNP flanking sequence were kept (approximately 2.8 million). These SNPs were loaded into the database and are intended to serve as potential biomarkers.

## Supporting Information

Figure S1 **The** **GeneNet Engine v0.9 Search Form.** The search form can be used to locate network modules by species, network name, module name, specific gene, functional annotation terms, traits, and simple topology.
(TIF)

Figure S2 **The GeneNet Engine** **Module Explorer.** Contains an interactive module viewer, a genome viewer with circular plots of the module, edge and node lists, functional enrichment report and trait selection tool to filter reports and views by specific genetic traits.
(TIF)

Figure S3 **Filter by Trait Tab of the Module Explorer.** Users can alter the module explorer to identify edges overlapping genetic features. Users can select features by trait name, genetic feature type, genetic maps (if applicable) and specify the amount of overlap required.
(TIF)

Table S1 **NCBI GEO microarray samples assigned to K-means clusters.**
(XLSX)
