# Supplementary figures and images for: A Systems-Genetics Approach and Data Mining Tool to Assist in the Discovery of Genes Underlying Complex Traits in Oryza sativa
Source: PLoS One. 2013 Jul 16;8(7):e68551. doi: 10.1371/journal.pone.0068551 (PMC3713027; doi:10.1371/journal.pone.0068551)

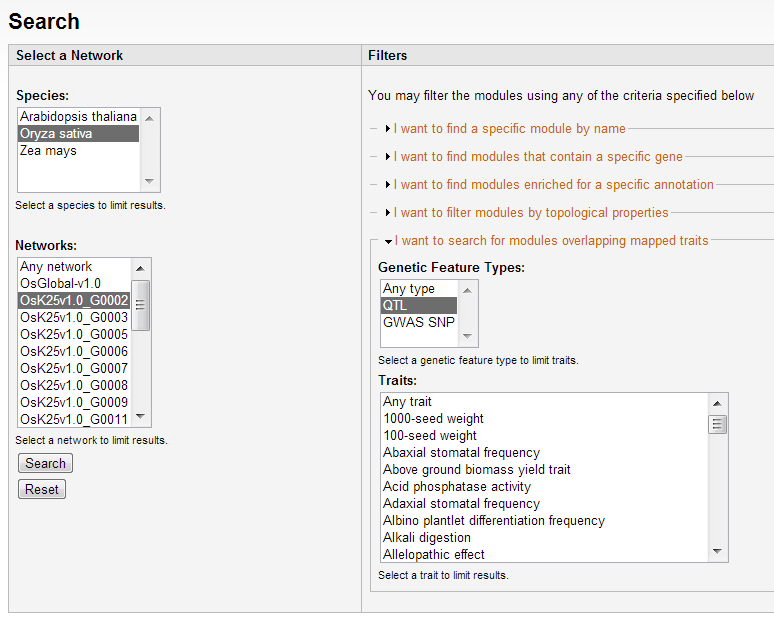

Supplement: Figure S1 — The GeneNet Engine v0.9 Search Form. The search form can be used to locate network modules by species, network name, module name, specific gene, functional annotation terms, traits, and simple topology. (TIF) [file pone.0068551.s001.tif]

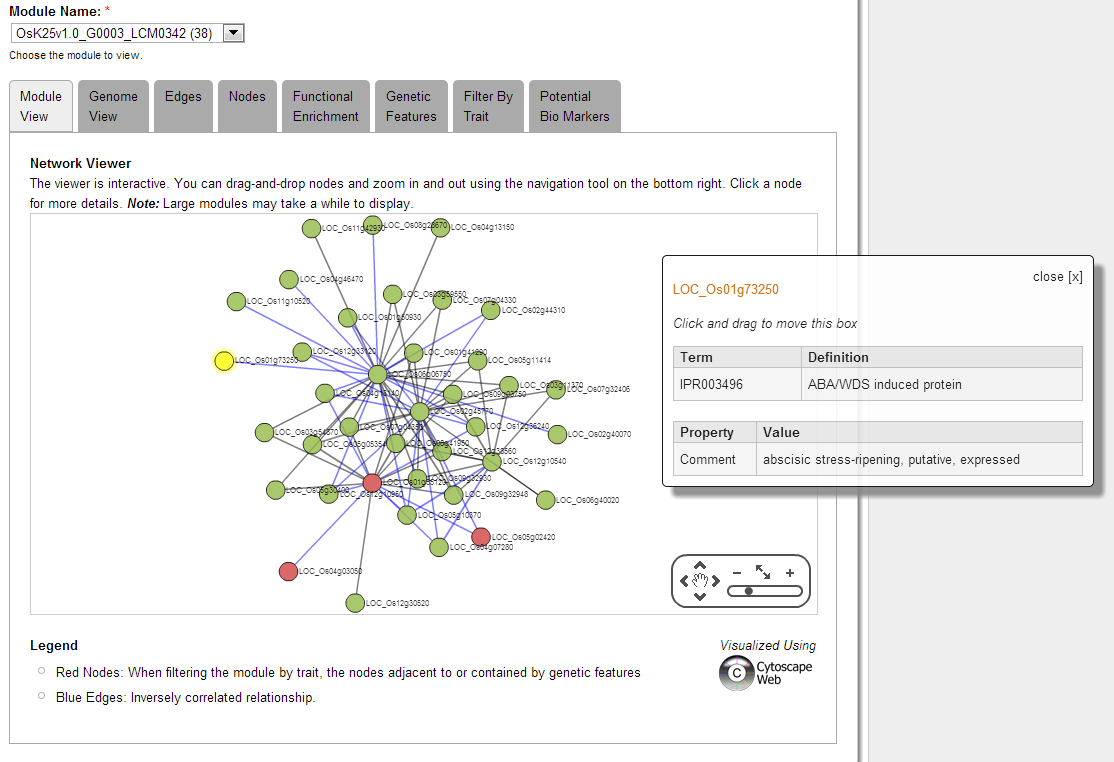

Supplement: Figure S2 — The GeneNet Engine Module Explorer. Contains an interactive module viewer, a genome viewer with circular plots of the module, edge and node lists, functional enrichment report and trait selection tool to filter reports and views by specific genetic traits. (TIF) [file pone.0068551.s002.tif]

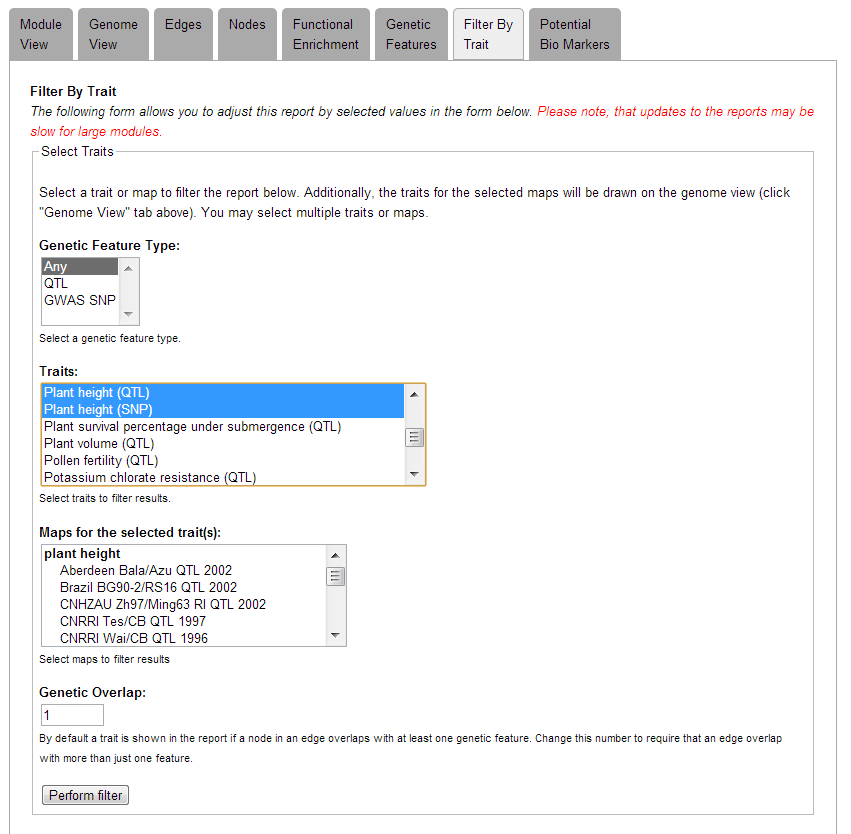

Supplement: Figure S3 — Filter by Trait Tab of the Module Explorer. Users can alter the module explorer to identify edges overlapping genetic features. Users can select features by trait name, genetic feature type, genetic maps (if applicable) and specify the amount of overlap required. (TIF) [file pone.0068551.s003.tif]
